# Distribution of syndecan-1 protein in developing mouse teeth

**DOI:** 10.3389/fphys.2014.00518

**Published:** 2015-01-15

**Authors:** Anna Filatova, Pierfrancesco Pagella, Thimios A. Mitsiadis

**Affiliations:** Division of Orofacial Development and Regeneration, Faculty of Medicine, Institute of Oral Biology, ZZM, University of ZurichZurich, Switzerland

**Keywords:** syndecan-1, tooth, incisor, odontoblast, ameloblast, tissue interactions, stem cells, stem cell niches

## Abstract

Syndecan-1 is a cell surface proteoglycan involved in the regulation of various biological processes such as proliferation, migration, condensation and differentiation of cells, intercellular communication, and morphogenesis. The extracellular domain of syndecan-1 can bind to extracellular matrix components and signaling molecules, while its intracellular domain interacts with cytoskeletal proteins, thus allowing the transfer of information about extracellular environment changes into the cell that consequently affect cellular behavior. Although previous studies have shown syndecan-1 expression during precise stages of tooth development, there is no equivalent study regrouping the expression patterns of syndecan-1 during all stages of odontogenesis. Here we examined the distribution of syndecan-1 protein in embryonic and post-natal developing mouse molars and incisors. Syndecan-1 distribution in mesenchymal tissues such as dental papilla and dental follicle was correlated with proliferating events and its expression was often linked to stem cell niche territories. Syndecan-1 was also expressed in mesenchymal cells that will differentiate into the dentin producing odontoblasts, but not in differentiated functional odontoblasts. In the epithelium, syndecan-1 was detected in all cell layers, by the exception of differentiated ameloblasts that form the enamel. Furthermore, syndecan-1 was expressed in osteoblast precursors and osteoclasts of the alveolar bone that surrounds the developing tooth germs. Taken together these results show the dynamic nature of syndecan-1 expression during odontogenesis and suggest its implication in various processes of tooth development and homeostasis.

## Introduction

Syndecan-1 is a member of a family formed by four proteoglycans (PGs) containing a C-terminal cytoplasmic domain, a well-conserved single-pass transmembrane domain, and a large N-terminal extracellular domain (Jalkanen et al., 1987; Sanderson and Bernfield, 1988; Saunders et al., 1989; Bernfield et al., 1992; Eriksson and Spillmann, 2012; Pap and Bertrand, 2013). The extracellular domain contains motifs for glycosaminoglycan (GAG) attachment, proteolytic cleavage, and cellular interactions (Salmivirta et al., 1991; Bernfield et al., 1992; Morgan et al., 2007; Eriksson and Spillmann, 2012; Pap and Bertrand, 2013). The binding of the intracellular domain of syndecan-1 to cytoplasmic proteins influences the dynamics of the cytoskeleton and promotes intracellular and membrane trafficking (Morgan et al., 2007). On the cell surface syndecan-1 strongly interacts with heparanase, which increases syndecan-1 shedding at the extracellular domain by stimulating protease expression (Kokenyesi and Bernfield, 1994; Morgan et al., 2007; Pap and Bertrand, 2013). In various tumorigenic conditions, syndecan-1 has been detected in the cell nucleus (Szatmári and Dobra, 2013; Kovalszky et al., 2014; Stewart and Sanderson, 2014).

Syndecan-1 is involved in the epithelial–mesenchymal interactions that take place during organogenesis, principally through its ability to bind growth factors and modulate their downstream signaling (Vainio and Thesleff, 1992; Perrimon and Bernfield, 2000). Syndecan-1 binds to a wide range of heparin-binding proteins such as Fibroblast Growth Factors (FGFs), Midkine (MK), and Hepatocyte Growth Factor (HGF) (Mitsiadis et al., 1995; Perrimon and Bernfield, 2000; Häcker et al., 2005; Teng et al., 2012). Signaling of these growth factors seems to be precisely controlled by regulatory loops involving syndecan-1 expression levels.

Tooth represents a suitable model system for studying epithelial–mesenchymal interactions and morphogenetic events during embryonic development (Thesleff et al., 1995). Sequential and reciprocal interactions between the oral epithelium and the underlying cranial neural crest-derived mesenchyme progressively transform the tooth primordia into complex mineralized structures with distinct cell types (Mitsiadis and Graf, 2009). Signaling molecules such as FGFs, MK, Bone Morphogenetic Proteins (BMPs), Wnt, and Sonic hedgehog (Shh) are involved in these interactions from the earliest stages of tooth initiation until the mineralization events (Mitsiadis and Luder, 2011). The epithelial-derived ameloblasts and the mesenchyme-derived odontoblasts are the highest differentiated dental cells that synthesize and secrete the organic components of the enamel and dentin, respectively.

A number of previous data have shown that syndecan-1 is expressed in the developing rodent molars and incisors (Vainio et al., 1989, 1991; Bai et al., 1994; Mitsiadis et al., 1995, 2008; Dias et al., 2005; Muto et al., 2007). Furthermore, a recent study has reported on the expression of syndecan-1 in developing human teeth (Kero et al., 2014). These findings have suggested that syndecan-1 is implicated in the subdivision of the mesenchyme into dental and non-dental territories, controls tooth morphogenesis and influences differentiation events. Although these earlier studies have shown syndecan-1 expression during precise stages of tooth development, there is no equivalent study regrouping the expression patterns of syndecan-1 during all stages of odontogenesis. For this reason, we have performed a systematic analysis of syndecan-1 protein distribution in the developing mouse molars and incisors, as well as in the alveolar bone.

## Materials and methods

### Animals and tissue preparation

Swiss and C57Bl/6 mice were used at embryonic and post-natal stages. The Veterinary Office of the Canton of Zurich (Switzerland) has approved the protocol working on mice (license Nr. 151/2014). Embryonic age was determined according to the vaginal plug (day 0) and confirmed by morphological criteria. Animals were killed by cervical dislocation and the embryos were surgically removed into Dulbecco's phosphate-buffered saline (PBS), pH 7.4. Dissected heads from the embryonic day 13.5 (E13.5) to the E18.5 mouse embryos and post-natal day 1 (PN1) to PN8 mouse pups were fixed in 4% paraformaldehyde (PFA) in PBS for 24 h at 4°C. After fixation the post-natal tissues were demineralized in acetic acid 0.1 N in 0.5% PFA in PBS for 5 days and then washed with PBS for 4 h. The heads were then dehydrated and embedded in paraffin wax. Seven micrometer serial sections were mounted on silanized slides and stored in air-tight boxes at 4°C until immunohistochemistry.

### Immunohistochemistry on tissue sections

A rat anti-mouse syndecan-1 antibody (281-2) was used (50 μg/ml). Preparation and characterization of this monoclonal antibody has been described earlier (Jalkanen, 1985). The specificity of this antibody has been verified previously by immunohistochemistry (Mitsiadis et al., 1995). A secondary goat antibody against rat IgG (1:500) was diluted in PBS and incubated at room temperature for 2 h.

Immunoperoxidase staining (ABC kit, Vector Laboratories, Burlingame, CA) was performed as previously described (Mitsiadis et al., 1995). Positive peroxidase staining produces red color on light microscopy. Replacement of primary antibody with normal rat serum served as a negative control.

## Results

### Syndecan-1 protein in developing molars

**Figure 1:**
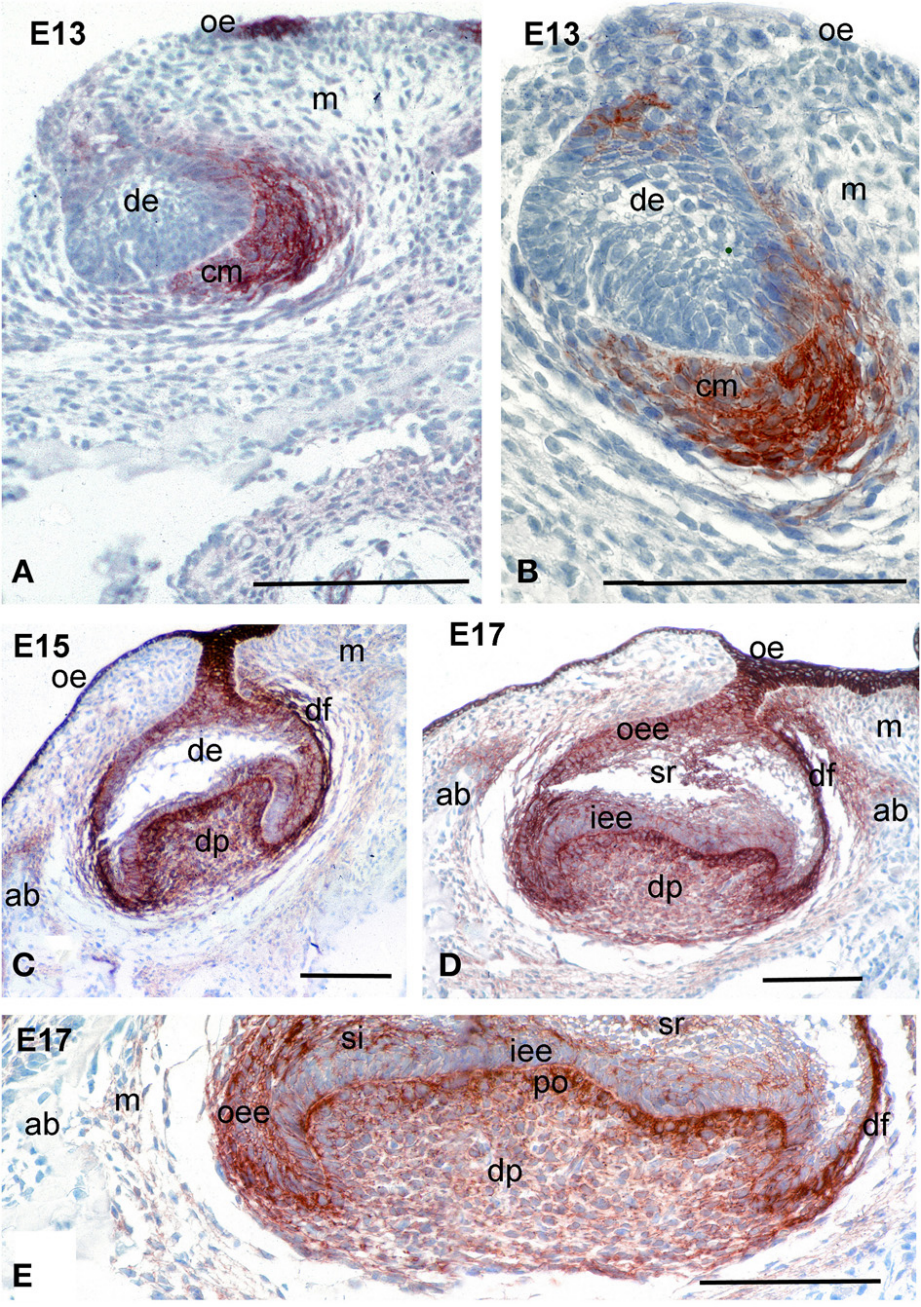
**Distribution of syndecan-1 protein in the developing first molars of mouse embryos**. Immunohistochemistry on frontal sections. Syndecan-1 staining (red color) in dental tissues during the bud **(A,B)**, late cap **(C)**, and early bell **(D,E)** stages of tooth development. **(E)** Higher magnification of the **(D)**. Abbreviations: ab, alveolar bone; cm, condensed mesenchyme; de, dental epithelium; df, dental follicle; dp, dental papilla; ED, embryonic day; iee, inner enamel epithelium; m, mesenchyme; oe, oral epithelium; oee, outer enamel epithelium; si, stratum intermedium; sr, stellate reticulum. Size bars, 200 μm.

At E13, the tooth epithelium forms a bud, around which the mesenchyme condenses. Syndecan-1 immunoreactivity was mainly detected on the surfaces of condensing mesenchymal cells that are close to the dental epithelium, whereas the staining in more peripheral mesenchymal cells was absent (Figures 1A,B). A faint and sporadic staining was also detected on the surfaces of some dental epithelial cells.

During the cap and early bell stages (E15–E17), syndecan-1 staining was seen on the surfaces of both dental epithelium and dental mesenchymal cells (Figures 1C–E). In the epithelium, intense reactivity was detected on cells of the outer dental epithelium and stellate reticulum, where the staining was weaker in cells of the inner dental epithelium and stratum intermedium (Figures 1C–E). In the mesenchyme, strong staining was observed in dental follicle cells, as well as in cells of the dental papilla in contact with the basement membrane that will differentiate into odontoblasts (Figures 1C–E). A weaker immunostaining was seen in fibroblasts of the dental papilla (Figure 1E).

**Figure 2:**
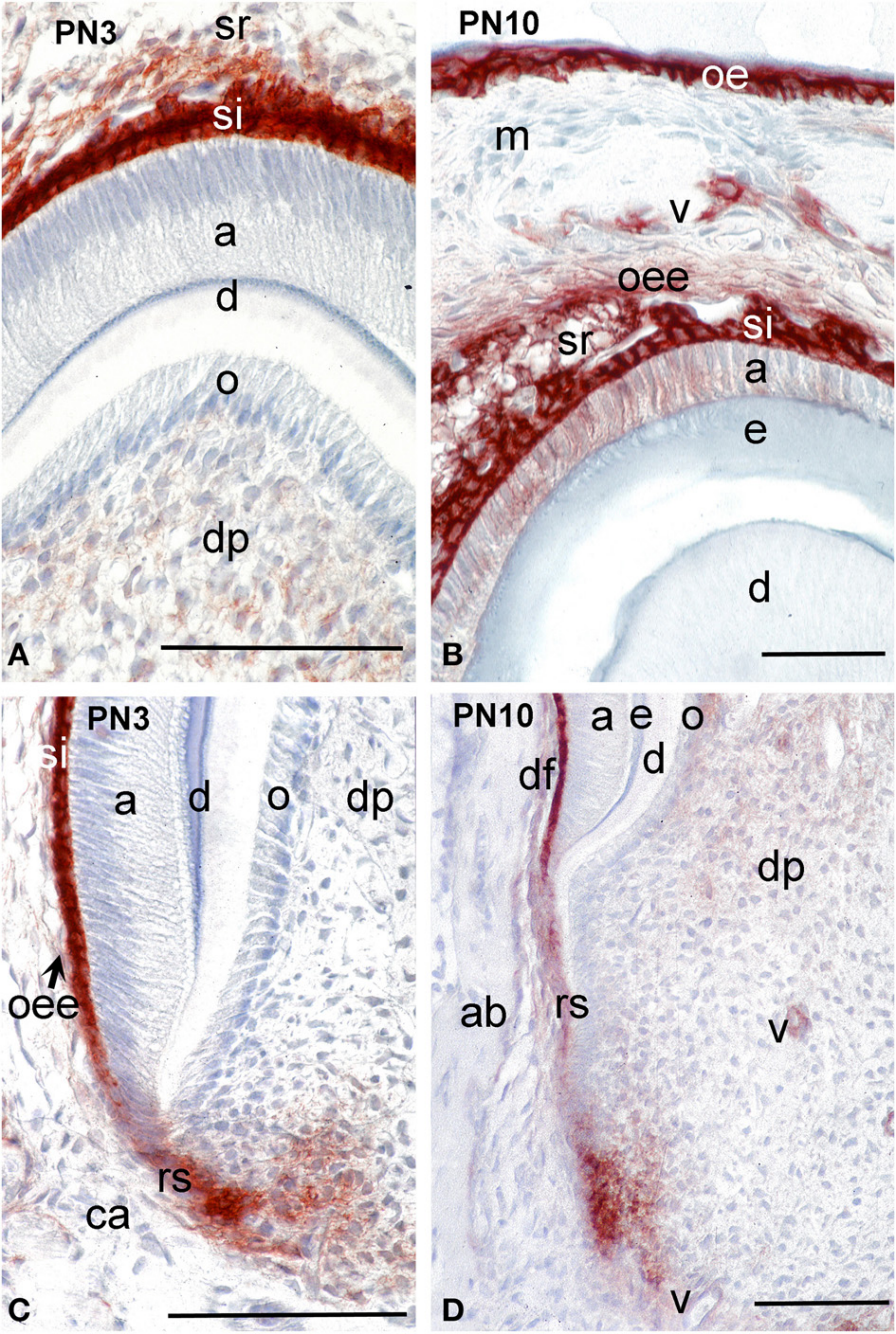
**Distribution of syndecan-1 protein in dental tissues of the developing first molars of mouse pups**. Immunohistochemistry on frontal sections. Sections through the crown **(A,B)** and root **(C,D)**. Syndecan-1 reactivity (red color) in tissues of developing tooth germs at post-natal day 3 (PN3) **(A,C)** and PN10 **(B,D)**. Abbreviations: a, ameloblasts; ab, alveolar bone; ca, cervical area; d, dentin; df, dental follicle; dp, dental pulp; e, enamel; m, mesenchyme; o, odontoblasts; oe, oral epithelium; oee, outer enamel epithelium; rs, root sheath; si, stratum intermedium; sr, stellate reticulum; v, vessels. Size bars, 100 μm.

During cytodifferentiation (PN3), syndecan-1 labeling was absent from odontoblasts and ameloblasts located at the tip of the cusps (Figure 2A) and the more apical areas (Figure 2C). However, the staining became very strong in cells of the stratum intermedium (Figures 2A,C) and in a lesser degree in stellate reticulum (Figure 2A). A faint reactivity was observed in the extracellular matrix of the dental pulp at the crown area (Figure 2A), while strong immunostaining was found in mesenchymal cells that are close to the forming epithelial root sheath at the cervical area (Figure 2C).

**Figure 3:**
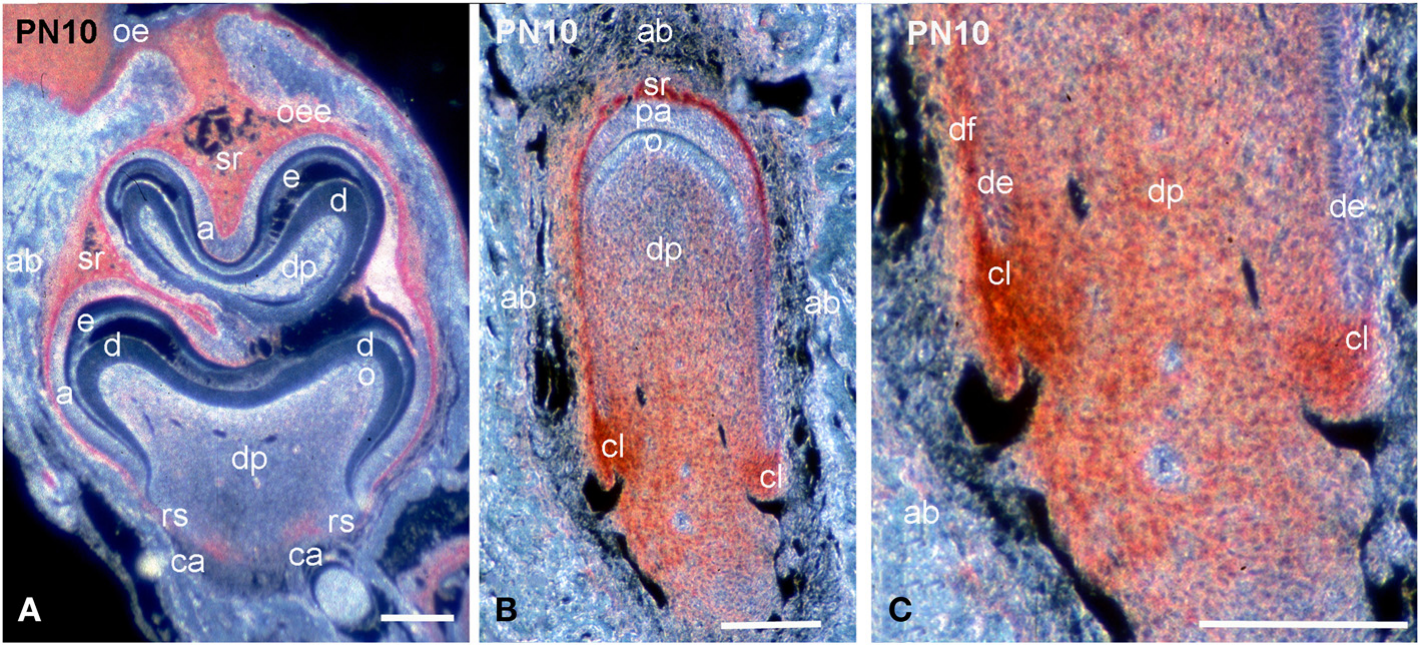
**Comparison of syndecan-1 protein distribution in first molars and incisors of PN10 mouse pups**. Immunohistochemistry on frontal sections. Sections through the molar **(A)** and incisor **(B,C)**. Syndecan-1 immunoreactivity in red color. **(C)** Higher magnification of Figure 3B. Abbreviations: a, ameloblasts; ab, alveolar bone; ca, cervical area; cl, cervical loop; d, dentin; de, dental epithelium; df, dental follicle; dp, dental pulp; e, enamel; o, odontoblasts; oe, oral epithelium; oee, outer enamel epithelium; rs, root sheath; sr, stellate reticulum. Size bars, 200 μm.

During mineral matrix deposition at P10, cells of the stratum intermedium, stellate reticulum and outer dental epithelium exhibited a very strong syndecan-1 reactivity (Figures 2B, 3A), while a faint labeling was detected in mature ameloblasts (Figure 2B). In the mesenchymal components of the tooth germ, moderate immunoreactivity was found in dental follicle cells and cells at the central part of the pulp (Figure 2D), whereas the staining persisted in dental pulp cells that are located near to the growing epithelial root sheaths (Figures 2D, 3A). A moderate syndecan-1 labeling was detected in epithelial cells forming the root sheath (Figure 2D) and in cells related to blood vessels (Figure 2D).

### Syndecan-1 protein in developing incisors

**Figure 4:**
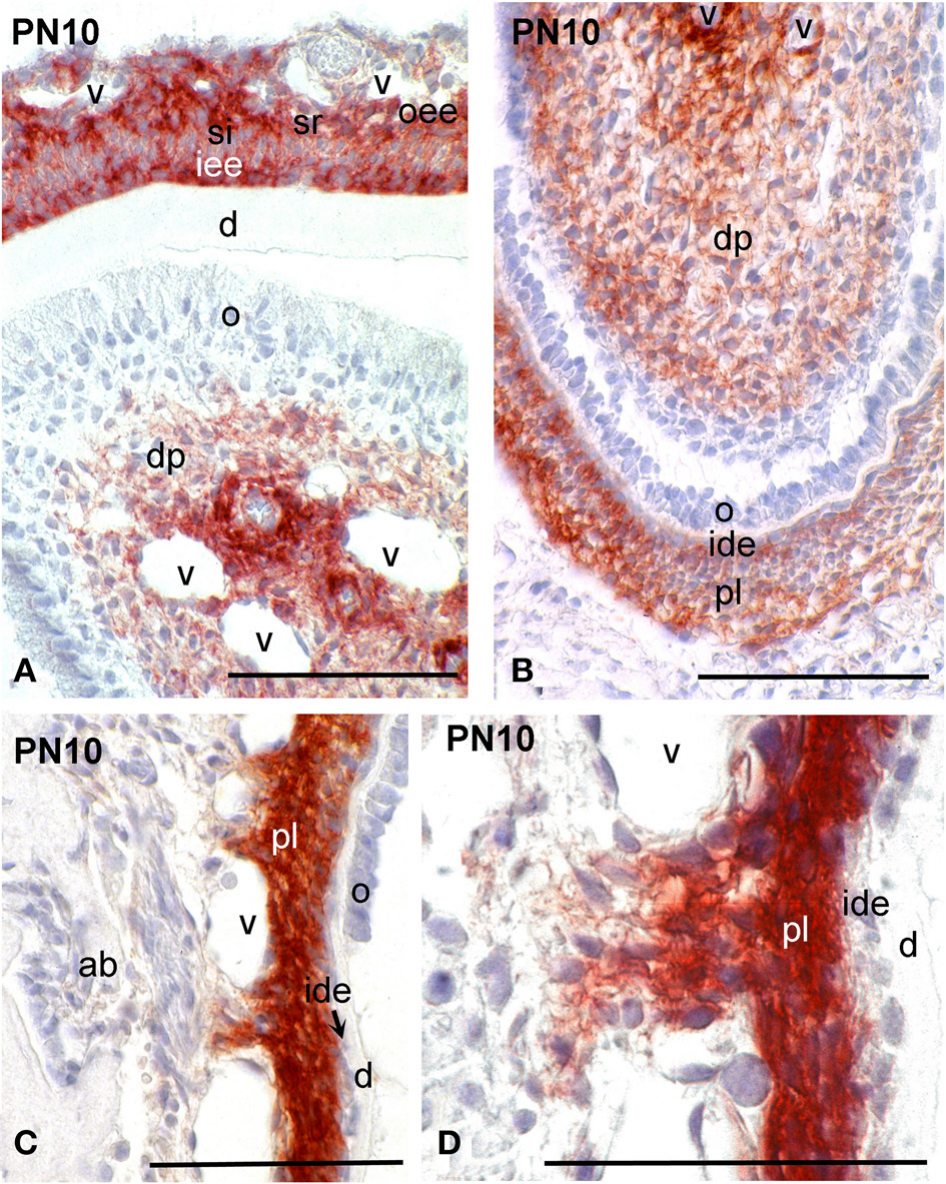
**Syndecan-1 protein distribution in tissues of PN10 mouse incisors**. Immunohistochemistry on frontal sections **(A–D)**. Syndecan-1 reactivity in red color. **(D)** Higher magnification of **(C)**. Abbreviations: ab, alveolar bone; d, dentin; dp, dental pulp; ide, inner dental epithelium; o, odontoblasts; oee, outer enamel epithelium; pl, periodontal ligament; si, stratum intermedium; sr, stellate reticulum; v, vessels. Size bars, 100 μm.

While at early stages of incisor development (E13.5) syndecan-1 presented similar distribution patterns to those observed in molar, at more advanced stages (E14.5–E16.5) an intensive labeling was detected in dental follicle mesenchymal cells (data not shown). A weak staining was observed in cells of the inner dental epithelium and stratum intermedium, whereas the immunoreactivity was absent from cells of the dental papilla mesenchyme (data not shown; Mitsiadis et al., 2008). From P1 to P8, syndecan-1 staining was detected on the surface of inner enamel epithelial cells and cells of the stratum intermedium, stellate reticulum and outer dental epithelium at the labial side of the incisor (enamel forming side), while in preameboblasts/ameloblasts the labeling was weak (Figures 3B, 4A). Strong syndecan-1 reactivity was observed in mesenchymal cells located at the central part of the pulp and the cervical loop region (Figures 3B,C, 4A,B), whereas the staining was absent in functional odontoblasts (Figures 3B, 4A). A similar pattern was seen in pulp cells located at the lingual side of the incisor (cementum forming side) (Figure 4B), where cells of the inner dental epithelium were negative for syndecan-1 (Figures 4C,D). Cells of the periodontal ligament exhibited very intense syndecan-1 immunoreactivity (Figures 4B–D).

### Syndecan-1 protein in the alveolar bone

**Figure 5:**
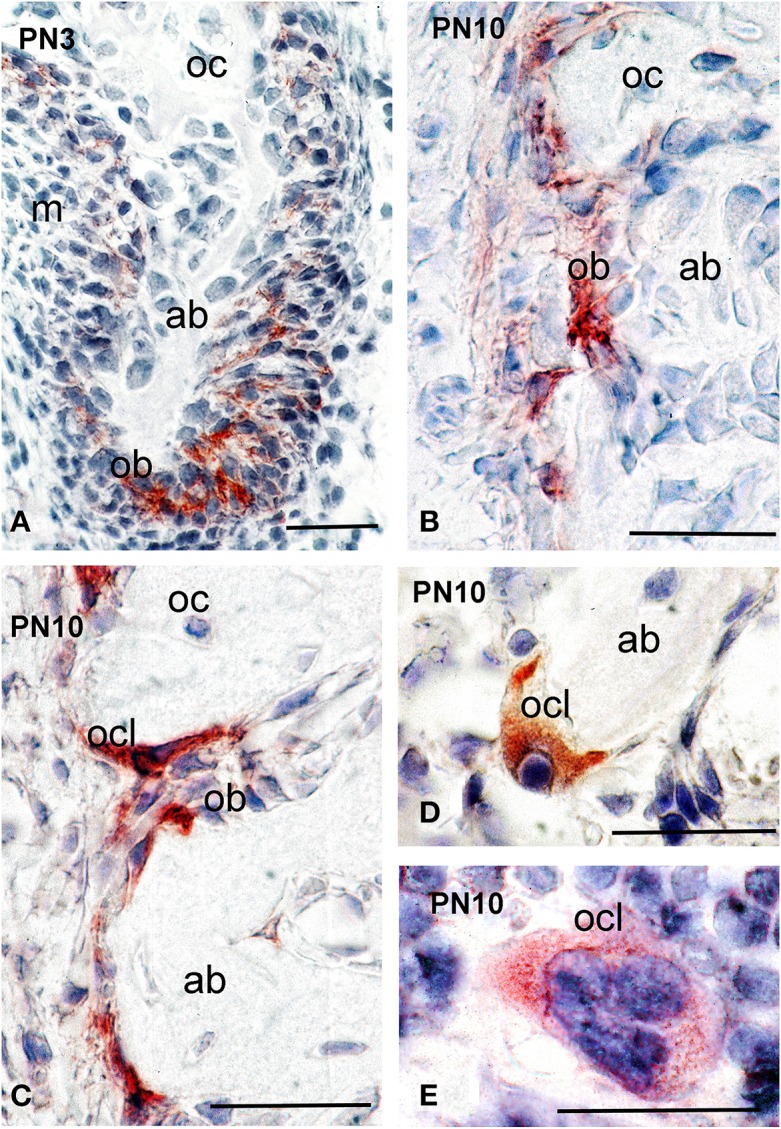
**Syndecan-1 protein distribution in alveolar bone of PN3 and PN10 mouse pups**. Immunohistochemistry on frontal sections **(A–E)**. Syndecan-1 reactivity in red color. Abbreviations: ab, alveolar bone; ob, osteoblasts; oc, osteocytes; ocl, osteoclasts. Size bars, 200 μm.

Alveolar bone formation and remodeling occurs during the mineralization stages of tooth development. At PN10, strong syndecan-1 immunoreactivity was detected in osteoblastic cells of the alveolar bone that surrounds the developing tooth germs (Figures 5A–C), as well as in osteoclasts (Figures 5C–E).

## Discussion

Transcription factors, secreted growth factors and extracellular matrix molecules provide the molecular signals for the epithelial–mesenchymal crosstalk that controls tooth development (Thesleff et al., 1995; Mitsiadis and Graf, 2009; Mitsiadis and Luder, 2011). The present study shows that syndecan-1 is distributed in distinct areas of the epithelium and mesenchyme during the early and late stages of odontogenesis. Syndecan-1 is involved in the signaling between epithelium and mesenchyme that is necessary for normal progression of tooth morphogenesis (Vainio et al., 1989). Syndecan-1 expression in the dental mesenchyme by E13.5 is associated with the transfer of the odontogenic potential from the epithelium to the mesenchyme (Vainio et al., 1989; Mitsiadis and Graf, 2009). Previous studies have shown that expression of syndecan-1 at these early stages of odontogenesis correlates with proliferation of mesenchymal cells (Vainio et al., 1991; Vainio and Thesleff, 1992). More recent studies have revealed that syndecan-1 also stimulates cell proliferation and migration in various pathological conditions (Iozzo and Sanderson, 2011; Teng et al., 2012). During the early bell stage of odontogenesis, syndecan-1 is distributed in both epithelial and mesenchymal compartments of the tooth germs. Once more, expression of syndecan-1 in the dental follicle mesenchyme is associated with intensive proliferative activity. In contrast, increased expression in odontoblast precursors (preodontoblasts) indicate that syndecan-1 may play an additional role in differentiation events (Hall and Miyake, 1995).

During the mineralization stage, the tooth crown achieves its definitive shape and the root formation begins. Functional ameloblasts that synthesize and secrete enamel matrix proteins express very little, if not any, syndecan-1 protein. Nevertheless, increased syndecan-1 expression is seen in other epithelial cell layers such as stellate reticulum and stratum intermedium. Decrease of syndecan-1 expression in dental pulp and dental follicle correlates with the progression of mesenchymal cell differentiation. However, syndecan-1 expression persists in mesenchymal cells located near to the growing epithelial root sheaths at the apical part of the tooth germ.

Rodent incisors are continuously erupting teeth, characterized by distinct zones of cell proliferation, differentiation, and maturation along their anterior–posterior axis (Mitsiadis et al., 2007; Mitsiadis and Graf, 2009). In incisors, syndecan-1 protein is synthesized in both epithelial and mesenchymal cells of its apical part (cervical loop region), preameboblasts, pulp fibroblasts, perivascular cells and cells of the periodontium. The presence of syndecan-1 in periodontal cells indicates its involvement in periodontium homeostasis and regeneration, as has been already suggested by previous studies (Worapamorn et al., 2000, 2001, 2002). Epithelial stem cells from the cervical loop generate transit amplifying progenitor cells that differentiate into all cell types of the incisor including the ameloblasts (Harada et al., 1999; Mitsiadis et al., 2007). Similarly, mesenchyme stem cells of the apical part of the incisor divide actively and generate the odontoblast progenitors (Mitsiadis et al., 2008). Syndecan-1 is found in the highly proliferative and specialized epithelial and mesenchymal compartments of the apical part of both molars and incisors as well as in perivascular pulp territories. These well-defined anatomical compartments of the teeth form distinct storage sites for stem cells that are defined as stem cell niches. Previous findings have indicated the existence of mesenchymal stem cell niches at the perivascular sites of the dental pulp (Shi and Gronthos, 2003; Lovschall et al., 2007; Mitsiadis et al., 2011). Similarly, many studies have shown the existence of epithelial and mesenchymal stem cell niches at the apical part of the teeth (Mitsiadis et al., 1999, 2011). Extracellular matrix molecules and adhesion molecules composing the dental stem cell niches may influence stem cell behavior (Mitsiadis et al., 2007). Syndecan-1 expression in these specialized dental territories suggests its implication in self-renewing, expansion, spread and progression of stem cells from the dividing and undifferentiated state to that of functional differentiated cells during tooth homeostasis and repair. Repair mechanisms that involve the activation and proliferation of stem cells in order to repopulate the dental injured tissues with the appropriate cell types (About and Mitsiadis, 2001; Mitsiadis and Rahiotis, 2004; Mitsiadis et al., 2011), may depend on syndecan-1 function. Indeed, it has been reported that heparan sulfate proteoglycans (HSPGs) affect proliferation, differentiation, and maintenance of the stem cells in developmental and repair processes (Häcker et al., 2005; Cool and Nurcombe, 2006; Bishop et al., 2007).

Signaling molecules such as Wnt, FGFs, Epidermal Growth Factor (EGF), and Notch are important regulators of stem cell fate and function (Häcker et al., 2005; Shaker and Rubin, 2010; Mitsiadis et al., 2011). It is likely that the diverse roles of these signaling molecules during odontogenesis are dependent on the simultaneous presence of syndecan-1, which is involved in the fine-tuning of their activity (Mali et al., 1993; Mitsiadis et al., 1995; Su et al., 2007). For example, the distribution of the syndecan-1 in the apical tooth areas correlates with the expression of FGF molecules (Harada et al., 1999, 2002; Mitsiadis et al., 2008), thus suggesting that syndecan-1 could modulate the effects of FGFs by either enhancing or reducing their activities (Mali et al., 1993; Bishop et al., 2007; Eriksson and Spillmann, 2012; Pap and Bertrand, 2013). Alternatively, syndecan-1 may act synergistically with these molecules to keep dental cells in an undifferentiated state (Mitsiadis et al., 2008).

Syndecan-1 can have distinct functions during proliferation and/or migration of cells in dental tissues. Several studies have shown that syndecans may be part of the MK and Heparin-Binding Growth Associated Molecule (HB-GAM or pleiotrophin) signaling during embryonic development (Mitsiadis et al., 1995; Nakanishi et al., 1997; Deepa et al., 2004). However, the patterns of syndecan-1, MK, and HB-GAM distribution differ as shown previously by comparative analysis of their expression in developing teeth (Mitsiadis et al., 2008), thus suggesting that syndecan-1 is only part of their signaling in precise developmental stages.

In post-natal life, syndecan-1 is found in both osteoblasts and osteoclasts of the alveolar bone. Syndecan-1 may enhance migratory or differentiation responses in osteoblasts and/or their progenitors. The present findings suggest that syndecan-1 may play a role in alveolar bone formation and remodeling. Indeed, syndecans are involved in skeletogenesis by regulating endochondral ossification and organizing the fine structure of the extracellular matrix (Pap and Bertrand, 2013).

The functional redundancy of syndecans in development has been long suspected due to their common ancestral origin and high sequence homology. Indeed, syndecan-1 knockout mice are healthy and do not exhibit any obvious organ abnormalities by the exception of mammary glands and corneal and skin re-epithelialization (Stepp, 2002; Liu et al., 2004). Furthermore, transgenic mice that overexpress syndecan-1 exhibit impaired wound healing due to the increased levels of shed syndecan-1 ectodomain at the injury site, where it acts as a dominant-negative regulator of syndecan-1 function (Elenius et al., 2004). Thus, syndecan-1 is involved in wound healing processes, and most notably in keratinocyte function and re-epithelialization.

**Figure 6:**
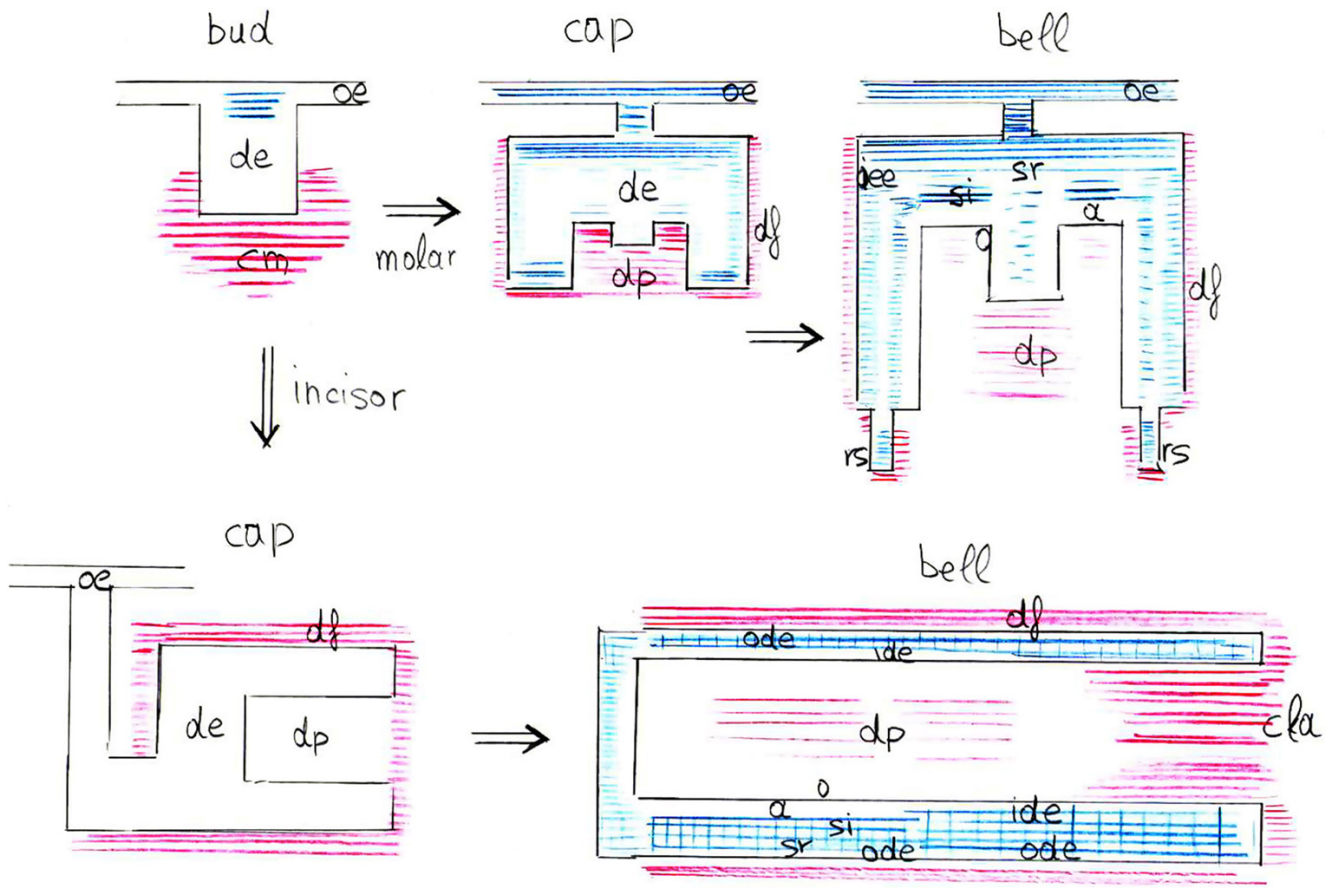
**Schematic representation of syndecan-1 protein distribution (blue color for epithelium, red color for mesenchyme) in the developing mouse molars and incisors (from bud to late bell stages)**. Abbreviations: a, ameloblasts; cla, cervical loop area; cm, condensed mesenchyme; de, dental epithelium; df, dental follicle; dp, dental pulp; ide, inner dental epithelium; o, odontoblasts; oe, oral epithelium; ode, outer dental epithelium; oee, outer enamel epithelium; rs, root sheath; si, stratum intermedium; sr, stellate reticulum.

In conclusion, the present results highlight the important and dynamic nature of syndecan-1 during tooth development and homeostasis (Figure 6). Syndecan-1 could be used as a marker of stem/progenitor cells that are activated during tooth repair processes. Syndecan-1 function on proliferative, migratory and differentiation events may greatly depend on the concomitant presence of signaling molecules.

### Conflict of interest statement

The Guest Associate Editor Dr. Claudio Cantù declares that, despite being affiliated to the same institution as author Prof. Thimios Mitsiadis, the review process was handled objectively and no conflict of interest exists. The authors declare that the research was conducted in the absence of any commercial or financial relationships that could be construed as a potential conflict of interest.
